# Health expenditures, environmental quality, and economic development: State-of-the-art review and findings in the context of COP26

**DOI:** 10.3389/fpubh.2022.954080

**Published:** 2022-10-26

**Authors:** Zhenjiang Xing, Xia Liu

**Affiliations:** ^1^School of Law, Shantou University, Shantou, China; ^2^School of Literature and Journalism, Guangdong Ocean University, Zhanjiang, China

**Keywords:** human health, governance, environmental quality, greenhouse gas, regulatory quality, human capital

## Abstract

There are numerous factors that affect human health. Among others, environmental degradation, bad governance, and extensive economic growth are regarded as more destructive for health-related issues. To explore the nexus of the said factors and extend the scholarly literature, the current study aims to analyze the influence of greenhouse gas (GHG) emissions, governance indicators, and gross domestic product (GDP) on human health expenditures—captured by domestic health expenditures and capital health expenditures. Specifically, this study contrasted variables including regulatory quality (RQ), rule of law (RL), GDP, GHG emissions, and human capital (HC) with that of human health expenditure. Covering the period from 1996 to 2020, this study uses time series specifications in the case of China, which is one of the largest pollution-emitting economies across the globe. The empirical results found that the long-run equilibrium relationship exists between the variables. For the long-run coefficients, this study utilizes the fully modified ordinary least square, dynamic ordinary least square, and canonical cointegration regression, suggesting that economic development and RQ are adversely affecting human health expenditure. However, GHG emissions, RQ, and HC significantly improve human health by increasing health expenditure in China. Based on the empirical results, policies are suggested regarding human health improvement, improved governance quality, and environmental sustainability. The study discusses the empirical conclusions and implications as per COP26 declarations.

## Introduction

Health is necessary and adequate for economic development. A healthy individual has a substantial role in economic progress (1) suggested that investing in the health sector increases economic growth and development in the country. Expenditures on health are a necessity not a luxury for any nation. Health expenses are considered an investment in human capital (HC) that significantly play role in economic growth (2). Healthcare expenditures whether public or private significantly contribute to the enhancement of health outcomes (3). The increasing global temperatures have also catastrophic impacts on the health of the people, which can only be accomplished when bigger countries like China step forward to mitigate these emissions. The healthcare community must play a significant role in mitigating greenhouse gas (GHG) emissions. According to the COP 26 Health program, international health organizations have established programs for the protection of people's health and planet earth. The initiative includes building resilient and sustainable healthcare systems. In the Conference of Paris 26, the discussion on health was the principal concern of many nations (4). Almost fifty countries around the world have sworn in COP 26 to expurgate healthcare emissions, which have a noteworthy role in overall disastrous GHG emissions. The increasing catastrophic emissions harm the human body causing inflammation, bone and kidney concerns, etc. The healthcare system in China has experienced some fundamental changes utilizing both public and private health and insurance programs. In China, almost 95% of the population has the least coverage of health insurance. For that reason, China has been ranked 144th in the world by the World Health Organization (WHO). With the largest population and highest gross domestic product (GDP) among developed economies, China has a relatively lesser number of doctors available, that is, one for thousand individuals (5).

The relationship between health expenses and economic growth is generally considered positive, but the connection is still a broadly discussed topic around the world. Attributable to (6), the determinants of health expenditure increase are macro and environmental variables or health policy variables. The rise in economic growth is essential for a lesser number of health outcomes. The improved health facilities have lesser health issues that can be achieved by increasing health expenses by local governments. Increasing the expenses for health negatively affects the health cases. Moreover, the food we eat, educational awareness, and lifestyle determine the quality and extent of someone's life (7). The health and economic growth causal association are helpful for several health policymakers for sustainable development of the country (8). The increase in income levels of people enhances their lifestyle and increases the welfare of the country. Nevertheless, the association is still ambiguous in some countries and also the condition is critical in China; there are still vulnerable groups due to the generalized system of preference (9).

The increasing environmental pollution has also deteriorated health outcomes. Health is affected by economic, social, and environmental factors. The deteriorating environment significantly affects people with increasing illnesses and diseases. The increasing carbon emissions cause air pollution, toxic chemical exposures, and noise pollution, which led to chronic respiratory and heart diseases in humans. These diseases have severe consequences, which affect later throughout life. The prevailing literature documents the substantial impact of carbon and GHG emissions on health expenditures (10, 11) also analyzed the association between health expenditure and carbon footprints in the United States of America (12) determined a significant relationship between health expenses and carbon emissions. In general, carbon emissions and health expenses are substantially linked with each other (13) recommended that ineffective budget allocation to healthcare momentously influences capital health expenditure (CHE). However, the deepened efforts can help achieve universal health coverage for enhancement of the health of individuals and policy regulations (14) expressed those regulatory institutions with effective implementations could be advantageous for the health of individuals.

The previous studies explored the association between economic growth, GHG emissions, and HC with health expenditures. In the prevalent literature, the studies usually exploited health expenditures as the independent variable to assess the economic growth of the country or carbon emissions as dependent variables or vice versa (8, 15–18). Therefore, the present study scrutinizes the role of economic growth, environmental emissions, and HC on two kinds of health expenditures in the case of China, a valuable input in the existing literature. Furthermore, the study is motivated due to poor healthcare systems in China, the country aims to advance the health systems besides making commitments to COP26 health programs. Hence, the study is inspired to assess the linkage in the case of China because the findings directly or indirectly influence health expenses and might be valuable in health policymaking.

The study has the following objectives. First, it aims to assess the influence of GDP, GHG emissions, regulatory quality (RQ), RL, and HC on the domestic government health expenditures (DGHEs) in the case of China. The second objective is to assess the impact of GDP, GHG emissions, RQ, RL, and HC on CHEs, a new input. To accomplish these objectives, the authors employed novel variables in two models as presented in Section 3. The study has employed novel variables such as RQ, RL, domestic health expenditures (DHEs), and CHEs to examine the aforementioned association in two models (1 and 2).

The study is significant in investigating the impact of economic growth, GHG emissions, RQ, RL, and HC on two different kinds of health expenditures. The increasing environmental emissions have deteriorated the health of people around the world. Hence, the study is substantial in the assessment of the said connection. The empirical findings of the present study would be significant in relevant policymaking or overhauling of the existent health policies. China is one of the biggest GHG emitters in the world; therefore, the present study and its outcomes could play important role in mitigating emissions and attaining a sustainable environment.

The study contributes to the literature in the following ways. First, the study is a pioneer in investigating the role of RQ and RL on health expenditures in the case of China. Attributable to increasing health and insurance concerns in the country, efficient use of institutions and regulations are beneficial in combating health apprehensions, specifically those that occur of increasing climate concerns. Second, the study utilizes the two kinds of health expenditures as dependent variables with two different specifications. The first is DGHEs which are typically operated by the government, and the second is CHEs or referred to as capital investments for healthcare/health. The analysis of these two innovative variables with other explanatory variables is a new and novel contribution that has not been explored before in any country. Third, the study contributes to the empirical literature by assessing the long-run causal relationship in the case of China from the period 1996 to 2020. The study employs cointegration analysis, the method of long-run estimates, quantile regressions, and pairwise Granger causality techniques for scrutinizing the linkage, which is a pioneering contribution to the prevailing literature.

The rest of the manuscript is organized as follows: Section 2 documents the literature about the variables under consideration. Section 3 deals with the data, model, and methodology of the research. Section 4 is about the results and discussions on the estimated results with interpretation. Lastly, Section 5 elaborates on the conclusion of the study.

## Literature review

The review of literature documents studies related to novel variables. The association between some novel variables has not been focused on before such as capital health investments, RQ, and RL, though this segment sheds some light on the linkage and empirical evidence of the variables prevailing in the literature.

Health is an essential and fundamental objective of a country (19) examined the effects of RL on health outcomes in data set of 96 countries. The empirical results demonstrated that sticking to the RL has a positive impact on health outcomes (3) observed that public and private health expenditures significantly contribute to the enhancement of health outcomes. Several studies have examined the impact and determinants of health disasters and health expenditure on economic growth and carbon emissions. For instance, (20) observed that economic growth, health expenditure, and HC significantly influence the outcomes of health. However, quite a few studies elaborated the influence and association between economic growth and carbon emissions' influence on health expenditures. However, some of them examined the causal association between GDP and health expenses (21) examined the positive relationship between GDP and health expenses. The increase in GDP increases the expenditures on health (22) scrutinized the panel causality analysis and depicted a bidirectional causal association between economic growth and health expenditures from GDP to health expenses and vice versa (8) explored the unidirectional association between healthcare expenditures toward economic growth in some nations such as Portugal, Korea, Ireland, and India because health expenses in these nations do not enough contribute to economic development. Hence, the role of expenses varies from country to country. However, some countries have represented bidirectional causality and unidirectional causality flowing from health expense to economic growth between the said variables (18) examined that health expenses significantly contribute to economic growth (Canada, Iceland, Norway, and Belgium) (23) also explained that maximum variation in health expenses occurs to changes in economic growth. (16) showed a causal association between health investment and the GDP of the economy. However, the existence of reverse causality exposed from GDP to health investment expenses affected the empirical findings.

Apergis et al. (15) explored the healthcare expenses on carbon emissions in the case of the United States from 1996 to 2009. The empirical findings demonstrated that carbon emissions have a stronger impact on health expenses, which led to an increase in health expenses (24) examined the health expenses and environmental emissions (carbon monoxide, sulfur oxide, etc.). The outcomes showed a positive and significant impact of emissions on health expenses in both periods (short and long runs) (11) also analyzed the association between health expenditure and carbon footprints in the USA. The cointegration results depicted a positive association between the said variables with unidirectional causality (25) examined significant associations (26) scrutinized the positive and bidirectional causal relationship between health expenditures and carbon emissions (27) analyzed the bidirectional causal relationship in the BRICS economies (17) depicted that increasing energy usage increases healthcare investments that led to higher emissions (28) determined the correlation between health expenditure and carbon dioxide emissions (29) observed a bidirectional association between healthcare expenses and carbon emissions.

A proper effective approach to regulation substantially impacts health expenditures (30, 31) suggested that various kinds of policies and regulations can help attain health expenditures. (13) suggested that inefficient budget allocation to healthcare has a significant impact on CHE. However, intensified efforts can help achieve universal health coverage for better health outcomes and regulations (14) expressed that regulatory institutions have a substantial impact on the health of individuals (32) demonstrated in the results that command and policy tools do not have enough significant associations with health outcomes as economic incentive policies (33) observed the influence of regulatory authority on ease of business (34) explored the influence of RL on the efficiency of microfinance. Likewise (35), examined the role of RL in capital market development (36) analyzed the behavior of RL on foreign investments and determined a positive association between them (37) examined the variable RL and suggested that sticking to the RL aids in mitigating carbon emissions (38) explained that the role of integrated policies and institutions has a significant impact on health expenditures (39) observed the RL principle's substantial influence on the expenses of health (40) examined the influence of institutional quality on the economic development of the country. The more effective quality of the institution is proportional to economic progress and health expenditures. The government must improve the institutions for better economic and health outcomes in the upcoming future (41) explored that improved institutional quality significantly enhances health outcomes by reducing mortality rates and increasing life expectancy and endorsing economic development. Azam et al. (42) examined the role of institutional quality in the process of sustainable development. The findings demonstrated a positive and constructive impact of institutional quality on sustainable development in different countries (43) examined the institutional quality variable on environmental pollution; however, the results depicted no impact on economic growth (44) determined a significant association between RL and economic performance and bank credit (45) represented that enhancing the quality of institutions has a substantial impact on eliminating carbon emissions. Azam et al. (42) scrutinized the role of institutional quality on environmental indicators. The findings indicated that there exists a constructive association between them.

Health is one of the components of HC that are prerequisites for economic development (46) quality of health is substantially essential and positive for productivity and economic progress (47) demonstrated in the findings that improvement in health expenditures significantly enhances the HC of the country (48) investigated HC in improving substantial impact on productivity and growth (49) observed that HC directly or indirectly affects the economic growth of the country (50) also examined the association between HC, economic growth, and health expenditures in the case of developing economies. The results depicted that health expenses and economic growth are substantially associated with HC. The overall general findings show that when HC is at high levels, the economic growth and health expenses significantly rise positively (51) demonstrated the importance of HC in the economic development of the country. Increasing the social capabilities enhances the social capabilities of economic growth (52) analyzed that health expenses and education have significant importance in the quality of HC (53) examined the effect of human health on the growth of the economy.

## Data and methodology

### Data and model construction

Following the study's objectives and previously mentioned literature, this study uses two dependent variables that indicate human health, specifically, the domestic general government health expenditure (DGHE measured as % of general government expenditure) and CHE (measured as % of GDP). In addition, the objectives of this study are to explore the influence of environmental degradation—captured by GHG(kt of carbon equivalent) emissions, and economic development—captured by GDP constant (US$ 2015 prices) on the dependent variables. Furthermore, this study also added RQ, RL, and HC) as control variables. Data for all these variables are collected from multiple sources including World Bank (2022) database. In this regard, World Development indicators provide the data on health expenditures and GHG emissions, whereas World Governance indicators provide the data for governance indicators. We employ the data from 1996 to 2020 for the case of China. Since China is recently dealing with the novel COVID-19 pandemic disease, it is crucial to analyze the specific association in the said country. Following (20), this study constructs the following two models:


(1) $$\begin{array}{l} \begin{array}{l} DHE_{t}\;=\;\alpha_{1}\;+\;\beta_{1}GDP_{t}\;+\;\beta_{2}GHG_{t}\;+\;\beta_{3}RQ_{t}\;+ \end{array}\\ \begin{array}{l} \;\beta_{4}RL_{t}\;+\;\beta_{5}HC_{t}\;+\;\varepsilon_{t} \end{array} \end{array}$$

(2) $$\begin{array}{l} \begin{array}{l} CHE_{t}\;=\;\alpha_{1}\;+\;\beta_{1}GDP_{t}\;+\;\beta_{2}GHG_{t}\;+\;\beta_{3}RQ_{t}\;+ \end{array}\\ \begin{array}{l} \;\beta_{4}RL_{t}\;+\;\beta_{5}HC_{t}\;+\;\varepsilon_{t} \end{array} \end{array}$$

The models reveal the intercepts and slopes *via* α's and β's, respectively. In addition, the subscript “*t*” indicates time series, and ε reports the random error term of the model.

### Estimation strategy

Nonetheless, there are numerous time series econometric approaches in existence that have been used in the literature. For instance, the literature uses quantile regression (20), Toda and Yamamoto causality test (22), Granger causality (8), error correction model (18), least square dummy variable and two-stage least square (16), among others. However, all the mentioned methods are selected based on the data specifications. Following the study of (3), this study also utilizes the fully modified ordinary least square (FMOLS), dynamic ordinary least square (DOLS), and canonical cointegration regression (CCR) approaches. In these approaches, the DOLS is regarded as a parametric approach, which is efficient when the number of parameter is finite. Furthermore, the FMOLS is a non-parametric approach, which is unlike linear regression, agnostic about the functional relationship between the outcome and the variables and, as a result, is immune to spurious regression error. Since the time series variables are mostly following the non-linear path of distribution, this study also intended to use quantile regression as a robustness test and is considered more effective and powerful in dealing the non-linear data.

Initially, this study evaluates the descriptive statistics including the mean, median, and range values. The range values constitute the minimum and the maximum values of the time series. Furthermore, this study also analyzes the SD of the variables, which generally refers to identifying volatility in the time series variable. All these specifications present the data in summarized form. Apart from the said specifications, this study scrutinizes the skewness and Kurtosis for data normality. To comprehensively analyze the distribution of time series, this study utilizes the (54) normality test, which considers skewness and excess Kurtosis simultaneously and presents statistical values. The said test proposes that the time series is normally distributed—considering the zero value of both the parameters. The statistical results of the said approach could be obtained as follows:


(3) $$\begin{array}{l} JB=\frac{N}{6}\left(S^{\;2}+\frac{{\left(K-3\right)}^{2}}{4}\right) \end{array}$$

Once the descriptive statistics are obtained, this study tends to identify the stationarity of the variables. Unlike the traditional unit root tests that examine only the unit root presence of the variables, such as the conventional Augmented Dickey–Fuller (ADF) and Phillip–Perron (PP) unit root tests, this test utilizes the breakpoint unit root test. As the former tests lack the property of indicating the structural break in time series, this test has more power in dealing with and identifying the structural break existing in the variable. The breakpoint unit root test assumes the presence of a unit root, yet the higher statistical values than the critical values could lead to the rejection of the null hypothesis. Since the stationarity of the variables is evident by the earlier unit root estimator. Therefore, it is important to examine the long-run equilibrium relationship between the variables. In this regard, this study utilizes the Johansen cointegration test. The Johansen test determines whether the time series variables are cointegrated. In particular, it evaluates the validity of a cointegrating connection using the maximum likelihood estimates. It is often used to determine the number of linkages and as an instrument to estimate such connections (55).

Using the Johansen cointegration test, the long-term correlation between variables was established. This allows the study to assess the impacts of each regressor, namely GDP, RQ, CRC, RL, and HC, on China's DGHE and CHE, respectively. Therefore, we need to utilize estimators that are impartial and appropriate. In this regard, as indicated by (56), this study also uses three long-term estimating approaches. Specifically, these approaches include the DOLS developed by (57), the FMOLS, and CCR proposed by (58). The earlier two estimators are two different approaches as one (FMOLS) is non-parametric, and the other one (DOLS) is a parametric approach. Since these estimators are more competent at addressing the endogeneity and serial correlation issues, their long-run predictions are reliable. In addition, the DOLS operator predicts the time series accurately since it handles the problem of non-stationarity. Both the FMOLS and DOLS are stated in the form of the following equation:


(4) $$\hat{\emptyset }=\left[\begin{array}{c} \alpha \\ \hat{\beta } \end{array}\right]\;=\;{\left(\sum_{t=2}^{T}Z_{t}\overset{'}{Z}t\right)}^{-1}\;\left(\sum_{t=2}^{T}Z_{t}y_{t}^{+}-T\left[\begin{array}{c} {\hat{\theta }}_{\text{'}}^{+}\\ 12\\ 0 \end{array}\right]\right)$$

From the above Equation (4), the FMOLS estimates could be obtained by utilizing $Z_{t}=\;(\overset{\prime }{{\overset{\prime }{X}}_{t},{\overset{\prime }{D}}_{t}})$. In utilizing the specification of FMOLS, the long-run covariance matrix is critical.


(5) $$y_{t}\;={\overset{\prime }{X}}_{t}\beta +{\overset{\prime }{D}}_{1}{{}_{t}}_{\gamma }{}_{1}+{\sum^{\text{​}}}_{j=-q}^{r}\Delta {\overset{\prime }{X}}_{t}{}_{+j}\sigma +v_{1t}$$

The equation above asserted the standard form of DOLS that further includes the cointegration regression by considering the leads and lags $\Delta {\overset{\prime }{X}}_{t}$ as a result of the orthogonal error term. This approach reveals that by the combination of *q* lags and *r* leads of various coefficients of regression, the long-term association could be detected between *e*_1*t*_ and *e*_2*t*_.

As mentioned earlier, the CCR estimation method is purely dependent on regression analysis. Nonetheless, this technique is cost-effective and crucial for eliminating the components of linear regression (59). Consequently, determining correct leads and lags, order is one of the greatest challenges for the approach under consideration. Generally, the CCR estimation techniques may be stated in the form of the following equation:


(6) $$\begin{array}{l} y_{t}^{*}={\overset{´}{\beta }}_{pq}z_{pqt}^{*}+\mu_{pqt}^{*} \end{array}$$

where the equation reveals the stationary transformation of both *y*_*t*_ and *z*_*pqt*_, respectively.

Although the above-mentioned specifications provide efficient inferences, still this study uses the quantile regression as a robustness test developed by (60). Since the variables under consideration follow an irregular path of distribution, this motivates the study to employ quantile regression as a robustness test, which is considered efficient in tackling abnormal data. In this study, the empirical statistics are obtained for four quantiles, i.e., 0.25, 0.50, 0.75, and 0.90. The general equation form of the said approach is given below for both Model 1 and Model 2, respectively.


(7) $$\begin{array}{l} \begin{array}{l} Q_{DHE_{it}}(\theta \;|\alpha_{i},\varphi_{t},X_{it})=\alpha_{i}+\varphi_{t}+\varphi_{1,\theta }GDP_{it}+\varphi_{2,\theta }GHG_{it}+ \end{array}\\ \begin{array}{l} \;\varphi_{3,\theta }RQ_{it}+\varphi_{4,\theta }RL_{it}+\varphi_{5,\theta }HC_{it}+\varepsilon_{it} \end{array} \end{array}$$

(8) $$\begin{array}{l} \begin{array}{l} \;Q_{CHE_{it}}(\theta \;|\alpha_{i},\varphi_{t},X_{it})=\alpha_{i}+\varphi_{t}+\varphi_{1,\theta }GDP_{it}+ \end{array}\\ \begin{array}{l} \varphi_{2,\theta }GHG_{it}+\varphi_{3,\theta }RQ_{it}+\varphi_{4,\theta }RL_{it}+\varphi_{5,\theta }HC_{it}+\varepsilon_{it} \end{array} \end{array}$$

As discussed earlier, the specific quantile (i.e., 0.25, 0.50, 0.75, or 0.90) is depicted by θ in the subscript for each explanatory variable.

Since the FMOLS, DOLS, CCR, and the quantile regression lack the assessment of the causal connection between the variables. Therefore, this study employs the pairwise (61) causality estimator. This test is efficient as it provides reliable estimates irrespective of the variable's integration, whether at level or the first difference. The pairwise Granger causality test proposed that there is no causal relationship exists between the variables.

## Results and discussions

### Results

**Figure 1:**
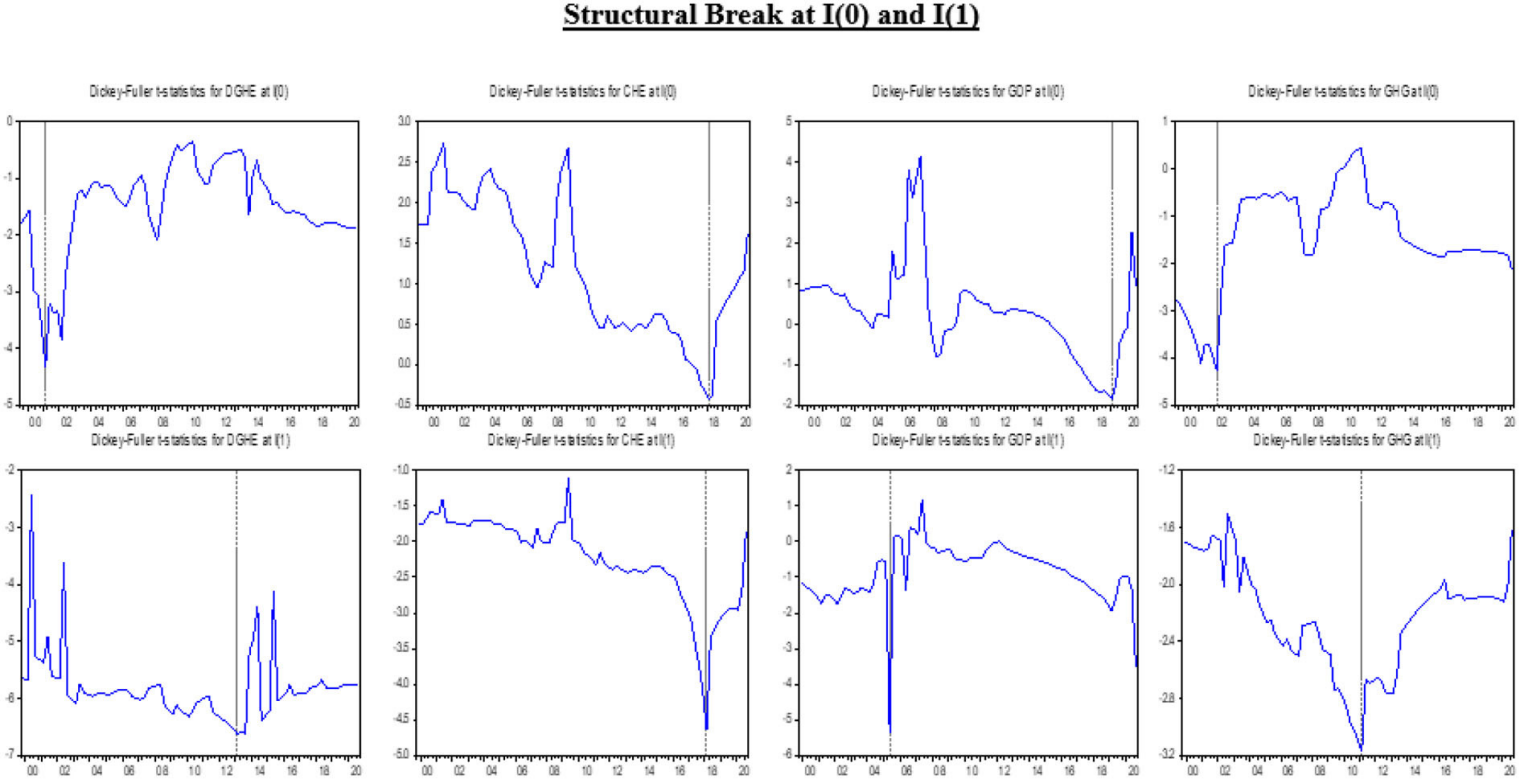
Graphical representation of structural breaks.

**Figure 2:**
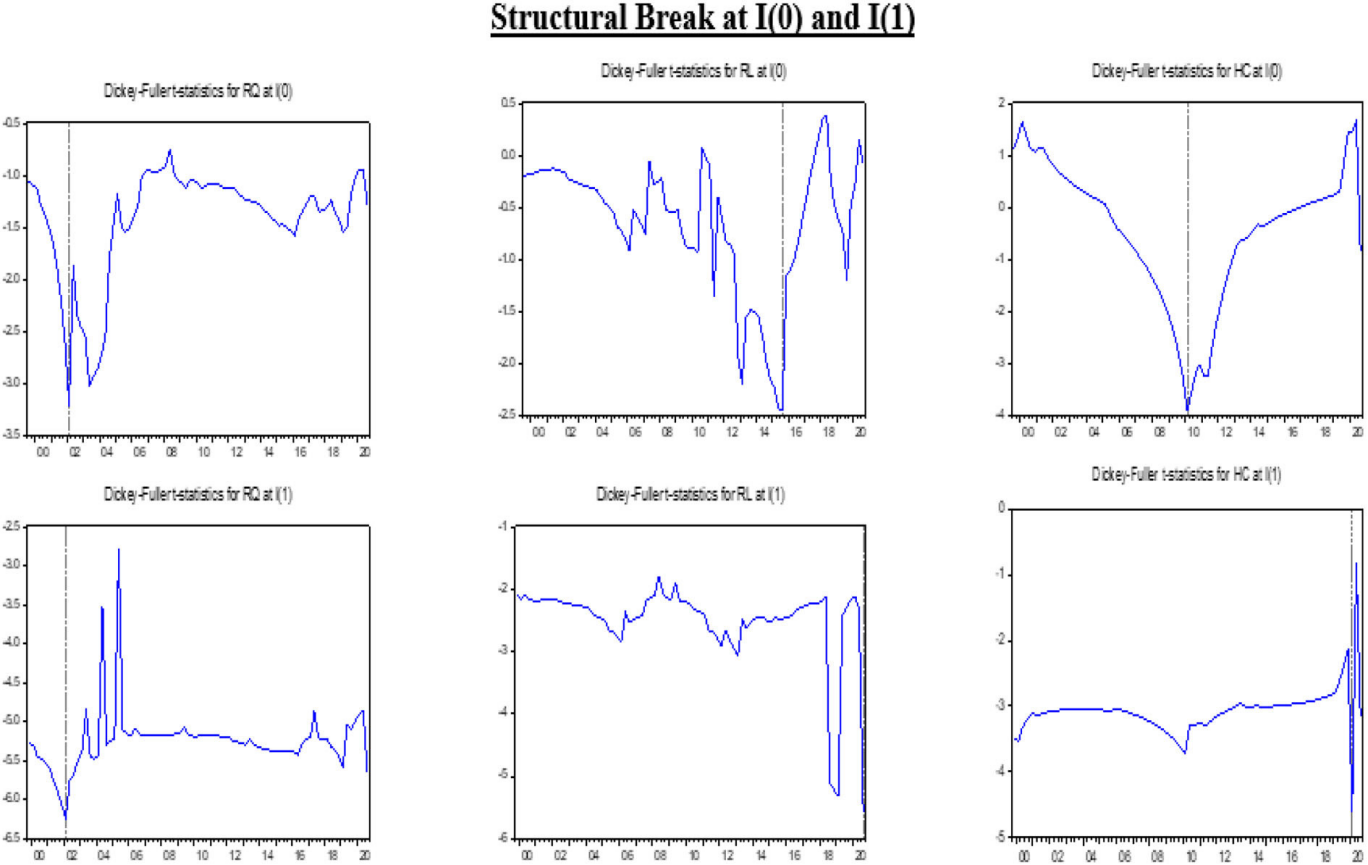
Graphical representation of structural breaks.

The results, their interpretations, and discussions are mentioned in this segment of the article. The descriptive statistics, the unit root test results with structural breaks and their graphical representation in Figures 1, 2 for both models (1 and 2), the long-run estimates from DOLS, FMOLS, and CCR (Models 1 and 2), and the quantile regressions to determine the robustness of both models besides the pairwise Granger causality outcomes are presented in this section.

#### Descriptive statistics

The mean and median values of the variables under study are nearly similar as presented in Table 1. The average values depict the balancing point of the data. The data spread from the average values are represented by values of SD. Skewness and Kurtosis document the data precision and symmetry. The values of skewness lie between +2 and −2 demonstrating the range of skewed distribution. The negative values show the presence of negative skewness. The values of Kurtosis in Table 1 lie between +7 and −7, except for RQ showing the peak of the distribution. The Jarque–Bera results are also significantly mentioned in Table 1.

**Table 1:** Descriptive statistics and normality results.

	**CHE**	**GDP**	**GHG**	**RQ1**	**RL**	**HC**	**DGHE**
Mean	0.371839	29.42606	15.91112	−0.262121	−0.442602	2.452907	7.671065
Median	0.338371	29.49678	16.01358	−0.248595	−0.482175	2.426106	7.971632
Maximum	1.064045	30.31422	16.37706	−0.080802	−0.059589	2.748987	9.626539
Minimum	0.010453	28.35354	15.27530	−0.770984	−1.034523	1.680717	3.714841
Std. Dev.	0.254018	0.637011	0.405781	0.100889	0.143371	0.170842	1.591712
Skewness	0.573370	−0.168651	−0.421724	−1.627691	0.235018	−0.673568	−0.697719
Kurtosis	2.826977	1.643562	1.568857	8.465884	5.600321	5.613638	2.249255
Jarque–Bera	5.603959	8.140402	11.49823	168.6392	29.09418	36.02448	10.46194
Probability	0.060690	0.017074	0.003186	0.000000	0.000000	0.000000	0.005348

#### Unit root test with structural breaks

The outcomes of the breakpoint unit root test are demonstrated in Table 2. All variables are insignificant at levels except DGHEs and GHG emissions. The structural break at the level occurs in Q1 of different years, whereas the structural break for RL appears in Q3 of 2015. At the first difference, the values are significant at 1% and 5% of the level of significance, except for GHG emissions. The negative values of the unit root depict the stronger presence of the unit root in the variable. The greater the negative value, the greater will be the unit root. The graphical presentation of structural breaks of each variable with respective years at level or first difference is presented in Figures 1, 2 The structural breaks report the abrupt changes that occur with time.

**Table 2:** Breakpoint unit root test.

**Variables**	**Augmented dickey**–**fuller statistics**			
	**I(0)**	**Structural break**	**I(1)**	**Structural break**
CHE	−0.419340	2018Q1	−4.638357^***^	2018Q1
DGHE	−4.324422^*^	2001Q1	−6.622531^***^	2013Q1
GDP	−1.834069	2019Q1	−5.373727^***^	2005Q2
GHG	−4.263341^*^	2002Q1	−3.172537	2011Q1
RQ	−3.214591	2002Q1	−6.242890^***^	2002Q1
RL	−2.454108	2015Q3	−5.583900^***^	2020Q3
HC	−3.901070	2010Q1	−4.606927^**^	2020Q1

*, ** and *** reports 10%, 5%, and 1% significance level.

#### Cointegration tests

The cointegration tests depict the correlation among the variables. The study applies the Johansen cointegration test. The Johansen test is applied after the series are determined stationary at the first difference and indicates the long-run associations of the variables. The findings of the test are presented in Table 3. The asterisk signs on the values indicate the rejection of the null hypothesis. The null hypothesis of the maximum eigenvalue (scalar) of no integration is rejected at a 5% level of significance in both models (1 and 2). The overall results depict the long-run associations of the study variables.

**Table 3:** Cointegration test.

**Johansen cointegration test**		
**Hypothesized No. of CE(s)**	**Eigen value (Model−1)**	**Eigen value (Model 2)**
None ^*^	0.511502^***^	0.477760^***^
At most 1 ^*^	0.294268^**^	0.347023^***^
At most 2	0.252125^*^	0.263131^*^
At most 3	0.165668	0.170330
At most 4	0.106401	0.098774
At most 5	0.053037	0.052826

*, ** and *** reports 10%, 5%, and 1% significance level.

#### Long-Run estimates

After the validation of the existence of a long-run relationship, the long-run estimates are analyzed in Tables 4, 5. The estimates are measured with DOLS, FMOLS, and CCR tests that assess the long-run relationship of the variables under consideration.

**Table 4:** Long–run estimates (Model 1).

**Variable**	**FMOLS**	**Std. error**	**DOLS**	**Std. error**	**CCR**	**Std. error**
GDP	−5.757^***^	0.401	−6.337^***^	0.325	−5.786^***^	0.388
GHG	10.117^***^	0.807	11.135^***^	0.662	10.851^***^	0.745
RQ	−3.143^***^	0.806	−3.730^***^	0.670	−2.729^***^	0.773
RL	1.454^*^	0.758	3.450^***^	1.074	3.249^***^	0.564
HC	6.595^***^	1.074	6.600^***^	0.894	2.568^***^	0.556
Adj. R^2^	0.965		0.981		0.921	

*, ** and *** reports 10%, 5%, and 1% significance level.

**Table 5:** Long–run estimates (Model 2).

**Variable**	**FMOLS**	**Std. error**	**DOLS**	**Std. error**	**CCR**	**Std. error**
GDP	−0.323^***^	0.051	−0.355^***^	0.039	−0.955^***^	−4.036
GHG	0.369^***^	0.103	0.455^***^	0.081	1.036^***^	4.078
RQ	0.136	0.104	0.090	0.091	−0.126	−1.199
RL	0.444^***^	0.099	0.404^***^	0.105	0.663^***^	5.757
HC	1.731^***^	0.125	1.587^***^	0.108	2.580^***^	7.358
Adj. R^2^	0.976026		0.989		0.979	

*, ** and *** reports 10%, 5%, and 1% significance level.

Table 4 shows significant values in all three tests for all variables with Model 1. For instance, GDP is significantly associated with DHEs in the long run. The coefficient of GDP indicates that a significant decrease in GDP increases DHEs. The positive value of the coefficient of GHG emissions indicates that increasing emissions significantly increase DHEs. RL is negatively associated with health expenses in the case of China, that is, increasing the stickiness of law has negative consequences on the health expenditures of the country. HC and RL has also a positive association with health expenditures in the long run. The increase in HC significantly increases the health expense of the country.

In Model 2, all variables are significantly associated with CHEs, except the RQ as shown in Table 5. RQ is positive but insignificantly related to CHEs in contrast with Model 1. GDP, GHG emissions, RL, and HC are significant for CHEs. Similarly, these variables are associated with DHEs, as depicted in the DOLS, FMOLS, and CCR tests of long-run estimates.

#### Test for robustness

After long-run estimates, a robustness test of quantile regressions is applied for the reliability of the model. It is also called an outlier-resistant model. Conventional linear regressions are affected by extreme values, whereas quantile regressions are less affected by extreme values. The quantile regressions analyze quarterly deflator accede to the robustness property. They are applied because conventional tests might not give correct results. It provides a predicted coefficient at specific quantiles. The empirical results of quantile regressions are displayed in Table 6 for Model 1 and Table 7 for Model 2. The robustness test is also referred to as the goodness-of-fit test that determines the reliability of the data.

**Table 6:** Robustness test for Model 1.

**Quantile regression**				
**Variable**	**Q**_**0.25**_	**Q**_**0.50**_	**Q**_**0.75**_	**Q**_**0.90**_
GDP	−2.755^***^	−3.190^***^	−2.415	−1.284
GHG	6.710^***^	7.077^***^	6.233^**^	5.264^***^
RQ	−2.027^***^	−1.860^***^	−2.440^***^	−2.560^***^
RL	−1.375	−1.319	−0.744	−1.147
HC	5.570^***^	5.982^***^	4.063	2.013
C	−33.006^***^	−26.79^***^	−31.143	−44.103^**^

Dependent variable is DGHE.

*, ** and *** reports 10%, 5%, and 1% significance level.

**Table 7:** Robustness test for Model 2.

**Quantile regression**				
**Variable**	**Q**_**0.25**_	**Q**_**0.50**_	**Q**_**0.75**_	**Q**_**0.90**_
GDP	0.633^***^	−0.519	0.781	1.820^***^
GHG	−0.380^***^	0.640	−0.834	−2.110^***^
RQ	−0.187	−0.108	0.526	0.872^***^
RL	0.294^*^	0.550^**^	0.180	−0.184
HC	−0.254^***^	1.749	0.125	−0.651^***^
C	−11.568^***^	1.370	−9.413	−17.772^***^

Dependent variable is CHE.

*, ** and *** reports 10%, 5%, and 1% significance level.

The majority of the quantiles of study variables are significant at a 10% level of significance, except for the RL, which is negative and insignificantly associated with DHEs as mentioned in Model 1. For CHEs, variables in the first (Q 0.25) and fourth quantiles (Q 0.90) are significantly associated with CHE, except RL. In general, the findings depicted the robustness of the model leading to examining the causal association among the variables under study.

#### Causality test

The causality analysis is employed to determine the causal relation of the long-run estimate variables. The quantile regressions could not provide causal associations; therefore, the Granger causality analysis is applied. In this study, the pairwise Granger causality test is utilized, and the results are portrayed in Table 8. The Granger tests reveal that some variable pairs are significant and causally associated. Out of 20 sets of variable pairs, only 11 pairs are bidirectionally or unidirectionally causally related. These are GDP ≠ DGHE, DGHE ≠ GDP, RQ ≠ DGHE, DGHE ≠ RQ, RL ≠ DGHE, DGHE ≠ RL, HC ≠ DGHE, CHE ≠ GDP, CHE ≠ RQ, RL ≠ CHE, and CHE ≠ HC.

**Table 8:** Causality test.

**Pairwise granger causality test**		
GDP ≠ DGHE	3.40583^**^	0.0374
DGHE ≠ GDP	6.70085^***^	0.0019
GHG ≠ DGHE	2.13329	0.1242
DGHE ≠ GHG	0.59394	0.5542
RQ ≠ DGHE	10.3500^***^	9.E−05
DGHE ≠ RQ	2.50615^*^	0.0871
RL ≠ DGHE	9.74690^***^	0.0001
DGHE ≠ RL	3.38316^**^	0.0382
HC ≠ DGHE	11.2743^***^	4.E−05
DGHE ≠ HC	0.41778	0.6597
GDP ≠ CHE	1.73835	0.1815
CHE ≠ GDP	2.70115^*^	0.0724
GHG ≠ CHE	1.20912	0.3031
CHE ≠ GHG	0.42091	0.6577
RQ ≠ CHE	0.36462	0.6955
CHE ≠ RQ	4.98557^***^	0.0088
RL ≠ CHE	3.35805^**^	0.0391
CHE ≠ RL	2.26887	0.1091
HC ≠ CHE	1.48947	0.2308
CHE ≠ HC	2.50332^*^	0.0873

Dependent variable is CHE.

*, ** and *** reports 10%, 5%, and 1% significance level.

Among these, GDP, RQ, and RL are bidirectionally associated with DHEs, whereas HC is unidirectionally associated with DHEs and CHEs running from HC to DHEs and CHEs to HC. GDP is also unidirectionally associated with CHEs flowing from CHEs toward GDP. Lastly, RL and RQ are significant with one-way directional associated with CHEs running from RL to CHEs and CHEs to RQ, respectively. The asterisks represent the significant values in the table. The general findings show that GDP, RQ, RL, and HC are significant with causal associations in the case of DHEs and CHEs. However, GHG emissions have insignificant causal associations in both models (1 and 2).

## Discussion

Evaluating the variables with descriptive statistics, the study employs unit root estimation with structural breaks. Determining the stationarity and unit root among the variables, Johansen tests were applied for examining the long-run relationships. Later, DOLS, FMOLS, and CCR tests were employed for scrutinizing the long-run guesstimates; then, quantile regressions as the goodness-of-fit test were utilized for reliable results. In the end, the causality analysis was held using the pairwise Granger causality tests for finding the causal linkage among the variable pairs under consideration.

Due to the inclusion of novel variables in the study, there was zero to no exact empirical evidence. However, the findings of the current study seem to be consistent with limited studies. The causality results of GDP are bidirectionally associated with DHEs and are consistent with the findings of (22, 50) stated that health and HC are significantly associated, which is in line with the present findings. The remaining pairs and their associations are novel to the available literature. The findings of the present research are also novel and innovative for economists and environmentalists. The GHG emissions have no such causal association with health expenses (DGHE and CHE). However, the coefficient of greenhouse shows a positive connection with health expenditures consistent with the outcomes (26). The positive associations between HC and health expenditure in the long-run estimation results are in line with those of (47). Although the rest is attributable to the novelty of the variables, there is no such study associated with the present findings that explain the causal and long-run relationships.

The study findings might be useful in revamping healthcare policies in China. As China is suffering from a healthcare crisis due to budget shortfalls and corruption in healthcare systems, increasing economic growth is important for health expenditures whether it is domestic or capital, and it is beneficial for the betterment of the systems. Furthermore, the RL, HC, and RQ of the institutions play a substantial part if efficiently utilized in enhancing health outcomes. These directly or indirectly influence health expenses, which are essential for overhauling the healthcare systems.

## Conclusion and policy implications

The study is noteworthy in examining the role of economic growth, GHG emissions, RQ, RL, and HC on two different kinds of health expenditures. The study has employed novel variables for the assessment of the aforesaid relationship. The increasing environmental emissions have impacted the health of people around the world. Therefore, the study is novel in scrutinizing human health, environmental quality, and economic development. The findings of the current study seem to be consistent with a few studies. GDP is two-way directionally related to DHEs, which are consistent with (22). Health and HC are significantly correlated (50). The coefficient of GHG is positively associated with health expenditures (26). However, there is no causal association found between GHG emissions and health expenditures (CHE and DHE). The positive associations between HC and health expenditure in the long-run estimation results are in line with those of 45. The remaining pairs with CHEs, RL, and RQ are novel to the existing literature. Therefore, there is scant empirical literature available. The discoveries of the present research are also novel and innovative for economists and environmentalists who describe the causal and long-run relationships. Furthermore, the empirical findings of the study would be significant in relevant policymaking or overhauling of the existent health policies. China is one of the biggest GHG emitters in the world; therefore, the present study and its outcomes could play important role in mitigating emissions and attaining a sustainable environment.

### Policy implications

Health is a public good. Government must take initiative on increasing health expenditures along with the adoption of renewed policies and overhauling the institutions for better health of the people. To advance the excellence of national health, the government needs to increase research and development in medical areas. The provision of basic health facilities along with subsidized insurance policies is the need of the hour and it must be provided. The improvement in government health expenses significantly reduces health outcomes. Providing basic health necessities and subsidies is also beneficial in limiting health issues in people. China must pay attention to environmental policies and adopt certain methods to mitigate harmful GHG emissions. Being the biggest emitter of carbon and GHG emissions, the country needs to adopt and implement certain policies for greener and cleaner energy. Furthermore, increasing the land with green vegetation and plants aids in reducing emissions by absorbing carbon dioxide. It is already suffering from a healthcare emergency due to budget deficits and bribery in healthcare systems. Hence, revamping and restructuring health policies are recommended. The increasing economic growth substantially affects health expenditures whether it is domestic or capital. Sustainable growth and RQ of the institutions are beneficial for the betterment of the healthcare systems. Furthermore, RL is a determinant of health. It has a significant role in health outcomes. Better laws are required for the apportionment of health expenditures for better health outcomes. Besides, there is a need for sustainable urban planning, enhanced and advanced health infrastructure, and capital health investments are essential for sustainable growth economically. COP 26 health program initiative is beneficial in eliminating emissions. It has accepted the global warming pact of preventing fossil fuel usage to improve the climate in the 2020s, China must work on these policies and their effective and immediate implementations.

### Limitations and future study recommendations

The study is limited to the case of China. However, for future purposes, the debate can be extended to other developed countries for in-depth analysis of the novel variables and global environmental policymaking. Furthermore, developing countries are more vulnerable to health concerns and climatic problems, and the present study can be replicated in order to scrutinize the causal linkage with the inclusion of other novel variables in developing economies that might help in assessing and revamping their health policies for imminent future. It is recommended to include environmental and economic variables for the analysis of health expenditures as a comparison of different countries.

## Data availability statement

The original contributions presented in the study are included in the article/supplementary material, further inquiries can be directed to the corresponding author.

## Author contributions

ZX: concept, data, analysis, and discussion. XL: analysis, preparing draft, editing, literature review, and conclusion. All authors contributed to the article and approved the submitted version.

## Funding

This work was supported by the National Social Science Foundation of China under Grant number 20BZZ103, the Research Institute of Local Government Development of Shantou University under Grant number 07421005, the Scientific Research Funds of Shantou University under Grant number STF21025, and the Humanities and Social Sciences Project of Guangdong Ocean University under Grant number C20135.

## Conflict of interest

The authors declare that the research was conducted in the absence of any commercial or financial relationships that could be construed as a potential conflict of interest.

## Publisher's note

All claims expressed in this article are solely those of the authors and do not necessarily represent those of their affiliated organizations, or those of the publisher, the editors and the reviewers. Any product that may be evaluated in this article, or claim that may be made by its manufacturer, is not guaranteed or endorsed by the publisher.
